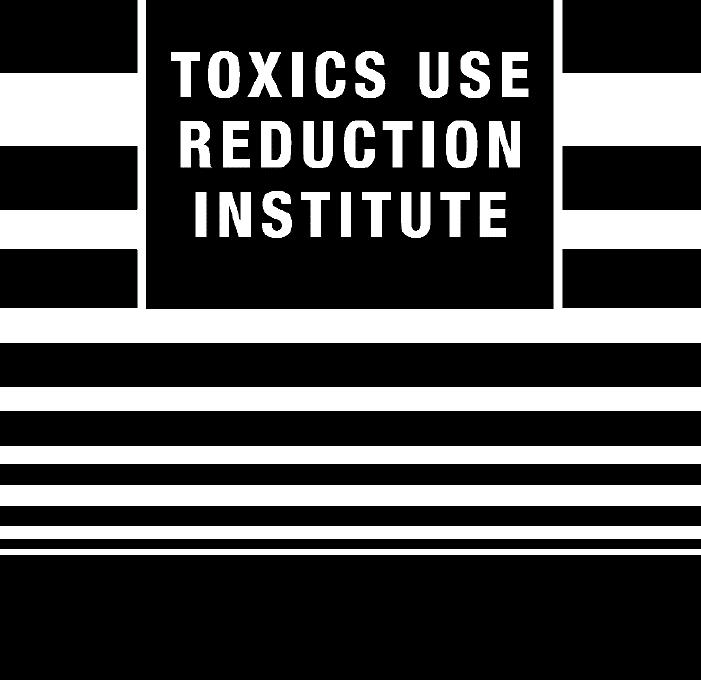# EHPnet: Massachusetts Toxics Use Reduction Institute

**Published:** 2006-12

**Authors:** Erin E. Dooley

The Massachusetts Toxics Use Reduction Institute (TURI), based at the University of Massachusetts Lowell, is the research and education arm of a three-part program established by the state’s 1989 Toxics Use Reduction Act (the program also includes regulatory and onsite consultation components). The TURI website at **http://www.turi.org/** describes the institute’s work in reducing the amount of chemicals used in manufacturing processes in Massachusetts, information that may be useful for those outside the state as well. By companies’ estimations, TURI helped reduce the amount of chemicals used by 41% since 1990 while at the same time helping companies improve their competitiveness. The institute reports that by-products from cleaning processes have been reduced during the same time period by 65%, and onsite chemical releases have dropped by 91%.

One of the newest TURI projects is the CleanerSolutions online database of surface cleaners, developed by the TURI Laboratory to help users identify safer cleaning products that perform as well as conventional products. A link to the CleanerSolutions website (**http://www.cleanersolutions.org/**) is found in the Online Tools section of the TURI homepage. The database contains 10 years’ worth of product testing information that is combined with health and environment indicators. The CleanerSolutions page on the TURI website also includes short interactive tutorials for using the database, a directory of other alternative cleaning online tools, and a section for vendors to propose items for inclusion in the database. Visitors can search the database for cleaning alternatives by either the contaminant to be removed, the solvent to be replaced, the type of equipment to be used, or the material to be cleaned. The TURI Laboratory conducts preliminary screening to determine potential risks of alternative chemicals based on global warming potential, ozone depletion potential, volatile organic content, flammability and reactivity, and acute toxicity.

The TURI website also has a Browse Topics page that catalogs information on the many projects the institute is working on. The categories listed include bio-based materials, cleaner production, competitiveness, corporate reporting, environmental management systems, nanotechnology, sustainability, and worker health and safety. The Corporate Reporting section includes an investor’s perspective on the value of sustainability reporting in socially responsible investing as well as case studies on how two companies have employed sustainability reporting or toxics use reduction in their activities.

Visitors can also find information on the TURI website using the links on the left side of the homepage to go to sections on Industry, Community, Government, TURI Training, the TURI Laboratory, Toxics Use Reductions Act Data, and the TURI Library. Available within the Research portion of the Industry section is the recently completed Five Chemicals Alternatives Assessment Study. This study was requested by the Commonwealth of Massachusetts to assess safer alternatives for lead, formaldehyde, perchloroethylene, hexavalent chromium, and di(2-ethylhexyl) phthalate. Along with the report is a Frequently Asked Questions page and documents related to the project. The Community section includes numerous fact sheets and other resources offering information on safer alternatives in areas such as auto maintenance and repair, construction, restaurant kitchen management, and household cleaning. This section also includes several sample policies, including one for schools and another for municipal pest management.

## Figures and Tables

**Figure:**